# Circulating Endocannabinoids as Diagnostic Markers of Canine Chronic Enteropathies: A Pilot Study

**DOI:** 10.3389/fvets.2021.655311

**Published:** 2021-05-26

**Authors:** Elettra Febo, Paolo Emidio Crisi, Sergio Oddi, Marco Pietra, Giorgia Galiazzo, Fabiana Piscitelli, Alessandro Gramenzi, Roberta Di Prinzio, Morena Di Tommaso, Nicola Bernabò, Tiziana Bisogno, Mauro Maccarrone, Andrea Boari

**Affiliations:** ^1^Faculty of Veterinary Medicine, University of Teramo, Teramo, Italy; ^2^European Center for Brain Research/Santa Lucia Foundation Istituto di Ricovero e Cura a Carattere Scientifico, Rome, Italy; ^3^Department of Veterinary Medical Sciences, Alma Mater Studiorum-University of Bologna, Bologna, Italy; ^4^Institute of Biomolecular Chemistry, National Research Council, Pozzuoli, Italy; ^5^Faculty of Bioscience, and Technology for Food Agriculture and Environment, University of Teramo, Teramo, Italy; ^6^Institute of Biochemistry and Cell Biology, National Research Council, Rome, Italy; ^7^Institute of Translational Pharmacology, National Research Council, Rome, Italy; ^8^Department of Biotechnological and Applied Clinical Sciences, University of L'Aquila, L'Aquila, Italy

**Keywords:** dog, endocannabinoid system, biomarkers, *N*-oleoylethanolamine, 2-arachidonoylglycerol, arachidonoylethanolamine, *N*-palmitoylethanolamine, chronic enteropathy

## Abstract

Chronic enteropathies (CEs) in dogs, according to the treatment response to consecutive trials, are classified as food-responsive (FRE), antibiotic-responsive (ARE), and immunosuppressive-responsive (IRE) enteropathy. In addition to this classification, dogs with loss of protein across the gut are grouped as protein-losing enteropathy (PLE). At present, the diagnosis of CEs is time-consuming, costly and sometimes invasive, also because non-invasive biomarkers with high sensitivity and specificity are not yet available. Therefore, this study aimed at assessing the levels of circulating endocannabinoids in plasma as potential diagnostic markers of canine CEs. Thirty-three dogs with primary chronic gastrointestinal signs presented to Veterinary Teaching Hospitals of Teramo and Bologna (Italy) were prospectively enrolled in the study, and 30 healthy dogs were included as a control group. Plasma levels of *N*-arachidonoylethanolamine (AEA), 2-arachidonoylglycerol (2-AG), *N*-palmitoylethanolamine (PEA), and *N*-oleoylethanolamine (OEA) were measured at the time of the first visit in dogs with different CEs, as well as in healthy subjects. Plasma levels of 2-AG (*p* = 0.001) and PEA (*p* = 0.008) were increased in canine CEs compared to healthy dogs. In particular, PEA levels were increased in the FRE group compared to healthy dogs (*p* = 0.04), while 2-AG was higher in IRE than in healthy dogs (*p* = 0.0001). Dogs affected by FRE also showed decreased 2-AG (*p* = 0.0001) and increased OEA levels (*p* = 0.0018) compared to IRE dogs. Moreover, dogs with PLE showed increased 2-AG (*p* = 0.033) and decreased AEA (*p* = 0.035), OEA (*p* = 0.016) and PEA (*p* = 0.023) levels, when compared to dogs affected by CEs without loss of proteins. The areas under ROC curves for circulating 2-AG (0.91; 95% confidence interval [CI], 0.79–1.03) and OEA (0.81; 95% CI, 0.65–0.97) showed a good accuracy in distinguishing the different forms of CEs under study (FRE, ARE and IRE), at the time of the first visit. The present study demonstrated that endocannabinoid signaling is altered in canine CEs, and that CE subtypes showed distinct profiles of 2-AG, PEA and OEA plasma levels, suggesting that these circulating bioactive lipids might have the potential to become candidate biomarkers for canine CEs.

## Introduction

Chronic enteropathies (CEs) in dogs are a group of heterogeneous disorders of undetermined etiologies, characterized by chronic persistent or recurrent gastrointestinal signs and histologic evidence of intestinal mucosal inflammation (1). According to the guidelines of the International Gastrointestinal Standardization Group, after exclusion of extra-intestinal, infectious or parasitic diseases, CEs can be subdivided retrospectively based on the response to different treatment into: food-responsive enteropathy (FRE), antibiotic-responsive enteropathy (ARE) and idiopathic inflammatory bowel disease (IBD). However, recently the use of the terms immunosuppressant-responsive enteropathy (IRE) or non-responsive enteropathy (NRE), rather than IBD, has been proposed (1, 2). For the latter, histopathologic evaluation of biopsy specimens is often required to confirm the presence of gut inflammation. However, histological assessment of inflammation can vary between pathologists, and agreement between pathologists remains poor (3, 4). In addition to this classification, dogs with loss of protein across the gut are typically grouped as protein-losing enteropathy (PLE), highlighting the more guarded prognosis of this particular form of CE compared to dogs with normal serum albumin concentration (5, 6).

At present, few diagnostic markers, such as the serum perinuclear anti-neutrophilic cytoplasmic antibodies (pANCA) and the fecal S100A12, showed an adequate accuracy in discriminating among the different forms of CE (7, 8). Thus, diagnosis and classification of the different canine CEs require the evaluation of clinical, laboratory and histological results, along with time-consuming and complex evaluations of the response to sequential therapeutic trials. In such cases the compliance of both the owner and the patient is often lacking (2). Although many non-invasive tests have been proposed, molecular biomarkers able to distinguish the different forms of CE remain as yet unavailable in clinical practice (7–9). In this context, it would be highly desirable to identify circulating molecules in blood, which are easy to measure with a simple blood test. They are able to simplify diagnosis and closely correlate with certain subsets (and/or with severity) of CE, which could serve as early biomarkers of disease onset and possibly predict the most appropriate treatment (9).

Newly discovered players in the homeostasis of intestinal functions are the endocannabinoids (eCBs). These are bioactive lipids produced in various organs, predominantly in the gastrointestinal tract, adipose tissue and the central nervous system. They act along with a set of receptors and enzymes that regulate their synthesis and degradation, the so called eCB system (10). The two most thoroughly studied eCBs are the arachidonic acid derivatives, *N*-arachidonoylethanolamine (AEA), and 2-arachidonoylglycerol (2-AG) (11). Other relevant “endocannabinoid-like” molecules involved in gut pathophysiology include: N-palmitoylethanolamine (PEA), which is an anti-inflammatory, analgesic, anti-convulsant and anti-proliferative agent; and the appetite-suppressor N-oleoylethanolamine (OEA) (12). The eCB system is highly expressed in the human and animal gut and maintains intestinal homeostasis, both under normal and pathophysiological conditions. It modulates many important functions, including the immune system, motility, sensation, secretion, inflammation and gut permeability, interaction with gut microbiota and gut brain fat-intake (13–18). The dysregulation of the eCB system may contribute to the development of several intestinal disorders. Several animal and human studies suggest an hyperactivation of eCB signaling during gut inflammation, either by increased receptor expression and/or by increased eCB levels (19–23). However, eCB system expression under different pathological conditions is often variable; therefore, it remains to be clarified how this signaling system regulates intestinal functions in healthy and diseased animals.

Recently, the immunohistochemical distribution of cannabinoid receptors in the canine gastrointestinal tract was reported (14). The expression of CB1 was documented in lamina propria and epithelial cells, and that of CB2 in lamina propria, mast cells, immunocytes, blood vessels, and smooth muscle cells of dogs (14). In addition, dogs suffering from chronic idiopathic large bowel diarrhea exhibited decreased expression of both CB1 and CB2 receptors in colonic mucosa when, compared with dogs in the control group (24). Against this background, the aims of this investigation were to determine plasmatic levels of AEA, 2-AG, PEA and OEA in healthy dogs and to evaluate their usefulness as biomarkers of CE in dogs, as well as their diagnostic ability in distinguish among the different forms of CE.

## Materials and Methods

### Animals

Dogs with primary chronic gastrointestinal signs presented to Veterinary Teaching Hospitals of the Universities of Teramo and Bologna, Italy, between January 2015 and September 2017, were prospectively enrolled in the study. Inclusion criteria were chronic (i.e., >3 weeks) gastrointestinal signs (e.g., vomiting, diarrhea, borborygmus, hyporexia, abdominal pain, and/or weight loss), as well as parasitological negativity of the copromicroscopic examination (i.e., direct fecal smear evaluation and zinc sulfate centrifugal flotation techniques) performed on 3 fecal samples. Common causes of chronic gastrointestinal signs such as exocrine pancreatic insufficiency or hypoadrenocorticism were excluded before patients were admitted to the study (i.e., complete blood count, serum chemistry panel, urinalysis, evaluation of basal cortisol or adrenocorticotropic hormone (ACTH) stimulation test if needed, canine Trypsin-like Immunoreactivity and abdominal ultrasound). SNAP canine pancreas-specific lipase (SNAP cPL^®^) assays were performed in those dogs that failed the dietary trial. The presence of extra-gastrointestinal disease or and recent (<1 month) antibiotic, pre- and probiotic and/or anti-inflammatory or immunosuppressive treatment excluded patients from the study. Dogs already in dietary management were not excluded from the study.

At admission, the Canine Inflammatory Bowel Disease Index (CIBDAI) (25), the Canine Chronic Enteropathy Clinical Activity Index (CCECAI) (6) and the nine-points body condition score (BCS) were obtained for each patient. Every dog underwent a 5-day course of fenbendazole 50 mg/kg orally once a day regardless to the fecal tests results. Moreover, measurements of serum folate, cobalamin and C-reactive protein (CRP) were also obtained.

Based on the diagnosis achieved considering the clinical response to sequential therapeutic trials, dogs affected by chronic enteropathy (CE) were retrospectively divided into 4 groups. The FRE group included patients with complete remission of the gastrointestinal symptoms within 3 weeks of dietetic trial, using a new exclusive diet (i.e., commercial novel protein diet, home-made novel protein diet or hydrolyzed protein diet); the ARE group included patients with partial or no response to dietetic trial, but complete remission of the gastrointestinal symptoms within 2–3 weeks of antibiotic trial with tylosin at dose of 15 mg/kg twice a day; the IRE group included patients that neither responded to diet nor to antibiotics and that went to complete remission on antinflammatory/immunosuppressive drugs such as prednisolone or budesonide.

Regardless of the diagnosis (i.e., FRE, ARE, IRE), CE patients were further included as non-PLE, i.e., those patients with albumin levels within reference interval, and PLE. i.e., those patients with low albumin concentration (<2 mg/dl) due to a severe loss of serum proteins into the intestine. For IRE and PLE dogs a histopathological analysis of multiple gastrointestinal endoscopic biopsies was available.

In order to assess the treatment success, dogs with CE were re-evaluated every 3 weeks after the initiation of treatment and the CIBDAI was recorded at every follow-up. As previously defined (26), the remission was considered complete if clinical signs resolved or the CIBDAI score reduced by ≥75% and maintained for a minimum of 6 weeks. In the ARE group a relapse within 6 weeks was considered acceptable and an additional course of tylosin was started if needed.

Healthy and normal weight (BCS 5/9) dogs, presented at Veterinary Teaching Hospitals for the annual check-up, pre-anesthetic evaluation before neutering or for blood donors selection were included in the control group. These patients had no clinical or pathological evidence of disease accordingly to an unremarkable history, physical examination and results of CBC, serum biochemistry and voided urine analysis. Recruited healthy dogs were on commercial maintenance diet and did not receive supplements and medications over the previous 4 months (except for regular preventive treatments for ecto- and endoparasites), as ascertained during the interview with the owners. Only dogs with an ideal BCS (i.e., 5/9) were enrolled as control group.

This study was performed with full informed consent of the owners and has been approved by the Committee on Animal Research and Ethics of the Universities of Chieti-Pescara, Teramo and Experimental Zooprophylactic Institute of AeM (CEISA; UNICHD12 N. 1168).

### Endocannabinoids Analysis

At the time of the first visit, each dog included in the study underwent blood sampling after a 12 h overnight fasting. In order to overcome the possible influence of circadian rhythm in eCBs secretion, all samples were collected between 8:00 a.m. and 12:00 p.m. Blood was collected in Vacuette K3-EDTA tubes, immediately centrifuges (10 min, 2000 rcf, 4°C), and plasma was immediately stored in 2 ml polypropylene tubes at −80°C until analysis (27, 28). The measurements of the eCBs were performed by the Endocannabinoid Research Group, at the Institute of Biomolecular Chemistry, National Research Council, Pozzuoli, Napoli, Italy. The study was conducted in a blind fashion where the operator was not given any information on the sample being analyzed. The plasma levels of 2-AG, AEA, PEA and OEA were determined in dogs with different chronic enteropathies (FRE, ARE, IRE and PLE) at the time of the first visit and in healthy subjects (control). The eCBs were analyzed and quantified after the extraction and purification procedures, generally within 3 days of sample collection.

Plasma samples were extracted in 5 volumes of chloroform/methanol/Tris–HCl 50 mM (2:1:1), containing 5 pmol of d8-AEA, 10 pmol of d4-PEA, d2-OEA and d5-2-AG (Cayman Chemicals, Ann Arbor, MI). The lipid-containing organic phase was dried down in a rotating evaporator and pre-purified by open-bed chromatography on silica gel columns eluted with increasing concentrations of methanol in chloroform. Fractions eluted with chloroform/methanol 9:1 by vol. (containing AEA, 2-AG, OEA, and PEA) were collected and aliquots were analyzed by isotope dilution-liquid chromatography/atmospheric pressure chemical ionization/mass spectrometry (LC-APCI–MS) using a Shimadzu high-performance liquid chromatography (HPLC) apparatus (LC-10ADVP), coupled to a Shimadzu quadrupole mass spectrometer (LCMS-2020) via a Shimadzu Atmospheric Pressure Chemical Ionization, APCI, interface. LC analysis was performed in the isocratic mode using a Discovery C18 column (15 cm × 4.6 mm, 5 μm) and methanol/water/acetic acid (85:15:1 by vol.) as mobile phase with a flow rate of 1 ml/min. MS detection was carried out in the selected ion monitoring mode using m/z values of 356 and 348 (molecular ion +1 for deuterated and undeuterated AEA), 384.35 and 379.35 (molecular ion +1 for deuterated and undeuterated 2-AG), 304 and 300 (molecular ion +1 for deuterated and undeuterated PEA), and 328 and 326 (molecular ion +1 for deuterated and undeuterated OEA). The levels of eCBs were then calculated on the basis of their area ratios with the internal deuterated standard signal areas, and their amounts were expressed as pmol/mL of plasma.

### Statistical Analysis

Data analysis was performed using statistical software (GraphPad Prism version 6.01, GraphPad Software, La Jolla California USA, www.graphpad.com). All data was evaluated using a standard descriptive statistic and reported as mean ± standard deviation (sd) or as median and range (minimum-maximum), depending on its distribution. Normality was checked graphically or using the D'Agostino Pearson test. Data of healthy dogs was inspected for outliers and a comparison among those of different reproductive statuses (i.e., entire males, neutered males, entire females and spayed females) was performed using the ANOVA or a Kruskall–Wallis test, and *post-hoc* tests (Student–Newman–Keuls test or Dunn test). Comparisons between control and CE groups as well as between CE dogs without PLE and PLE dogs were performed using the unpaired *t*-test or the Mann-Whitney test, while a comparison among the subgroups were done using the ANOVA or a Kruskall–Wallis test and *post-hoc* tests (Student–Newman–Keuls test or Dunn test). A regression analysis was used to evaluate the correlation between eCB values and CIBDAI or CCECAI, as well as the correlation between eCBs and BCS, CRP, folate and cobalamin.

For those eCBs that showed statistically significant differences in the comparison between the different CE groups, the sensitivity (Se), specificity (Sp), and negative and positive likelihood ratios (–LR and +LR) at different cut-off point and receiver operating characteristic (ROC) curves were used to assess the accuracy of the eCBs in distinguishing the different chronic enteropathies. A *P*-value < 0.05 was considered significant.

## Results

### Clinical Characteristics of the Study Population

Dogs affected by CE were 23 males (two castrated) and 10 females (four spayed). Purebreds (*n* = 27) were Zergpinscher (*n* = 3), Corso (*n* = 2), German Shepherd (*n* = 2), Maltese (*n* = 2), Appenzeller Sennenhund (*n* = 1), Australian Shepherd (*n* = 1), Beagle (*n* = 1), Belgian Shepherd (*n* = 1), Boxer (*n* = 1), Bull Terrier (*n* = 1), Cocker Spaniel (*n* = 1), Dachshund (*n* = 1), Dobermann Pinscher (*n* = 1), Dogue de Bordeaux (*n* = 1), English Pointer (*n* = 1), Golden Retriever (*n* = 1), Jack Russell Terrier (*n* = 1), Labrador Retriever (*n* = 1), Pug (*n* = 1), Rottweiler (*n* = 1), Scottish Shepherd (*n* = 1), and Siberian Husky (*n* = 1); six Dogs were mixed breeds.

Dogs belonging to the control group (Supplementary Table 1) were 11 males (three castrated) and 19 females (nine spayed), with 23 purebreds and seven mixed breed dogs. In the control group the represented breeds were Jack Russell Terrier (*n* = 5), Golden Retriever (*n* = 3), English Bulldog (*n* = 2), English Setter (*n* = 2), Siberian Husky (*n* = 2), Alaskan Malamute (*n* = 1), American Pitbull Terrier (*n* = 1), Beagle (*n* = 1), Belgian Shepherd Dog (*n* = 1), Bernese Mountain Dog (*n* = 1), Border Collie (*n* = 1), Corso (*n* = 1), Dobermann Pinscher (*n* = 1) and Rottweiler (*n* = 1).

Among CE dogs, 10 were diagnosed as FRE (seven males, one female and two spayed females), nine as ARE (eight males and one spayed female), 14 as IRE (six males, two castrated males, five females, and one spayed female), and five of them had a PLE (two males, one castrated male, one female, and one spayed). In tested dogs (i.e., nine ARE and 14 IRE) SNAP cPL^®^ were visually normal.

Age and body weight of dogs belonging to the different study groups are shown in Table 1. Supplementary Table 2 presents the comparison of age and bodyweight among the study dogs.

**Table 1:** Median age and body weight of the study dogs.

**Study groups**	**Age (in months)**	**Body weight (in kg)**
Control (*n* = 30)	60 (6–102)	21.3 (5–59)
FRE (*n* = 10)	21 (12–36)	30 (7–53)
ARE (*n* = 9)	60 (12–117)	19.6 (2.1–42)
IRE (*n* = 14)^*^	35 (8–144)	12.9 (2.8–45)

* *IRE group includes five PLE dogs. Ranges in brackets*.

Dogs affected by CE had lower BCS values when compared to healthy ones (*p* < 0.0001).

In the comparison among the different CE groups, no differences were observed in BCS, CIBDAI, CCECAI, CRP, folate and cobalamin (Table 2).

**Table 2:** Median values, with minimum and maximum in brackets, of Body Condition Score (BCS), Canine Inflammatory Bowel Disease Activity Index (CIBDAI), Canine Chronic Enteropathy Clinical Activity Index (CCECAI), serum concentrations of C-reactive protein (CRP), folate and cobalamin in dogs with Food-responsive Enteropathy (FRE), Antibiotic-responsive Enteropathy (ARE), and Immunosuppressive-responsive enteropathy (IRE).

**Variable**	**FRE**	**ARE**	IRE^*^	***P*-value**
BCS	4 (4-5)	4 (2–6)	3.5 (2–5)	0.12
CIBDAI	5 (3–8)	2 (1–6)	5 (1–11)	0.07
CCECAI	5 (3–10)	3 (1–6)	5 (1–14)	0.09
CRP (0–1 mg/dL)^**^	0.20 (0.02-3.21)	0.98 (0.16–1.65)	0.20 (0.01–5.65)	0.68
Folate (6.5–11.5 μg/L)^***^	6.78 (2.44–5)	11.10 (3.72–25.00)	6.57 (2.14–24.00)	0.31
Cobalamin (250–730 ng/L)^†^	395 (183–1,000)	547 (318–1,000)	269 (150–855)	0.07

* *IRE group includes five PLE dogs*.

** *available in 4/10 FRE dogs, 3/9 ARE dogs, 11/14 IRE dogs*;

*** *available in 8/10 FRE dogs, 7/9 ARE dogs, 14/14 IRE dogs*;

† *available in 8/10 FRE dogs, 7/9 ARE dogs, 14/14 IRE dogs*.

Complete haemato-biochemical analysis did not show any particular alterations in the different groups, except for the expected hypoalbuminemia in subjects with PLE.

Dogs belonging to the IRE and PLE groups underwent gastroduodenoscopies. In addition, an ileoscopy and colonoscopy were performed in all of the five dogs with PLE and in three IRE dogs at the discretion of the attending clinician. Biopsies were considered adequate in all dogs. Moderate to severe lymphoplasmacellular infiltrates were found in the all of the duodenum biopsies associated with moderate gastric fibrosis in four dogs without PLE and in two with PLE. Lymphangiectasia was detected in three dogs without PLE and in three with PLE, and was associated with multifocal atrophy of the villi in the latter animals. Mild to severe lymphoplasmacellular inflammation was observed in 7/8 dogs while in one dog ileal biopsy was within normal limits. Mild to moderate lymphoplasmacellular infiltrate was detected in the colon biopsies of eight dogs and was associated with fibrosis in three of them.

### Analysis of Endocannabinoid Levels

Dogs affected by CEs, independent of the diagnosis, had higher plasma levels of 2-AG (*p* = 0.001) and PEA (*p* = 0.008) compared to controls, whereas levels of AEA (*p* = 0.659) and OEA (*p* = 0.659) were similar between CE and healthy dogs (Table 3).

**Table 3:** Mean with standard deviation (SD) and median with minimum and maximum (min-max) of healthy dogs excluded suspected outliers.

**eCBs**	**Sample size**	**Outliers**	**Mean (±SD) excluded suspected outliers**	**Median (min-max) excluded suspected outliers**	**Lower 95% CI of mean**	**Upper 95% CI of mean**
2-AG	30	4	4.0 (±1.7)	3.4 (1.6–8.7)	1.625	2.02
AEA	30	1	1.8 (±0.5)	1.7 (0.9–2.8)	3.29	4.70
PEA	30	0	28.4 (±15.4)	24.5 (7.8–65.9)	22.67	34.19
OEA	30	0	56.4 (±25.9)	52.1 (9.2–121.1)	46.77	66.19

*The 95% confidence interval of the means were also reported. 2-AG, 2-arachidonoylglycerol; AEA, N-arachidonoylethanolamine; PEA, N-palmitoylethanolamine; OEA, N-oleoylethanolamine*.

**Figure 1:**
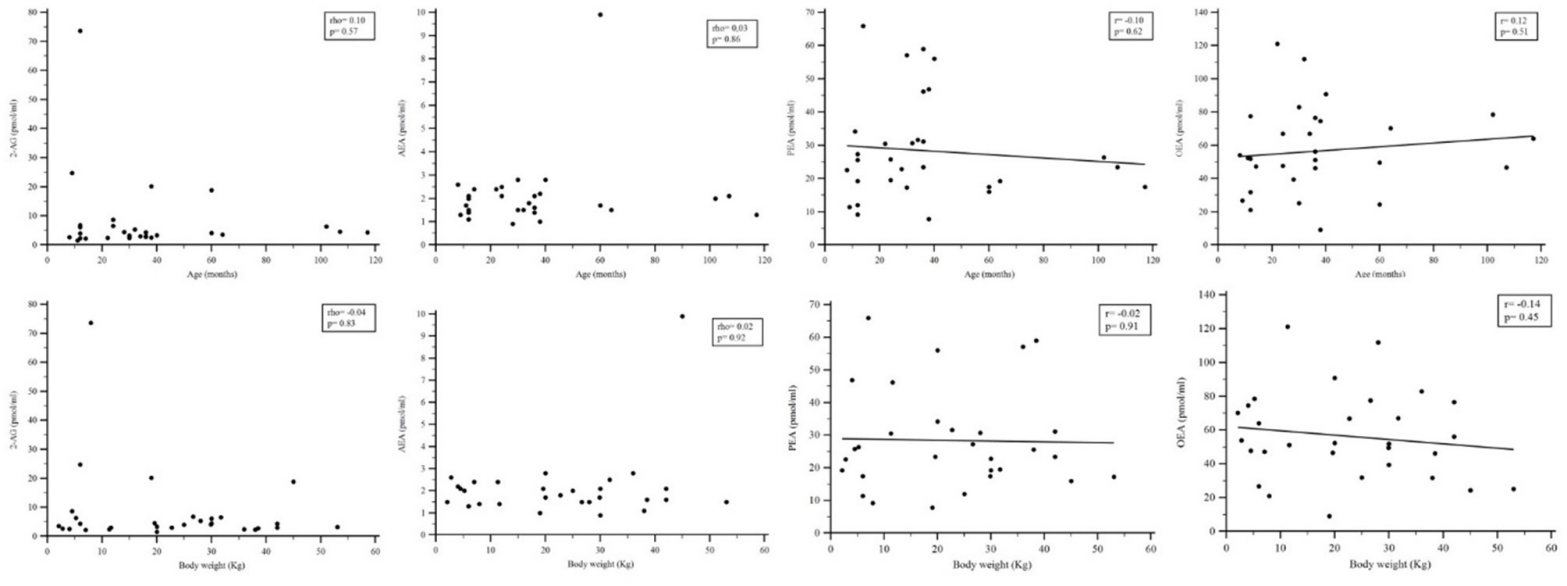
Rank correlation analysis comparing plasmatic concentrations of endocannabinoids and age or bodyweight in healthy dogs. 2-AG, 2-arachidonoylglycerol; AEA, N-arachidonoylethanolamine; PEA, N-palmitoylethanolamine; OEA, N-oleoylethanolamine.

Values of circulating eCBs in plasma of healthy dogs are shown in Tables 3, 4 (Supplementary Figure 1). The comparison of eCB levels in control dogs according to the gender and the neutering status did not show statistically significant differences (Table 5). Among healthy dogs, no correlations between levels of circulating eCBs and bodyweight or age were observed (Figure 1).

**Table 4:** Median values, with minimum and maximum in brackets, of plasma endocannabinoids (eCBs) in dogs with chronic enteropathy (CE, *n* = 33) and control group (*n* = 30).

**eCBs**	**CE group**	**Control group**	***P*-value**
2-AG	10.7 (1.9–220.6)	4.1 (1.6–73.6)	*0.001*
AEA	1.7 (0.9–4.8)	1.8 (0.9–9.9)	0.659
PEA	40.5 (8.0–120.3)	24.5 (7.8–65.9)	*0.008*
OEA	61.7 (2.8–167.0)	52.2 (9.2–121.1)	0.659

*2-AG, 2-arachidonoylglycerol; AEA, N-arachidonoylethanolamine; PEA, N-palmitoylethanolamine; OEA, N-oleoylethanolamine. Italic denotes significance*.

**Table 5:** Median values, with minimum and maximum in brackets, of plasma endocannabinoids (eCBs) inin healthy females (*n* = 10), spayed females (*n* = 9), males (*n* = 8), and castrated males (*n* = 3).

**eCBs**	**Males**	**Males castrated**	**Females**	**Female spayed**	***P*-value**
2-AG	2.8 (1.6–73.6)	4.1 (2.2–6.6)	3.2 (2.3–24.8)	5.3 (2.5–20.2)	0.266
AEA	1.9 (1.3–2.8)	2.4 (1.7–2.5)	1.5 (0.9–2.8)	2.1 (1.0–9.9)	0.139
PEA	29.0 (9.2–57.1)	19.5 (17.5–65.9)	26.4 (11.4–59.0)	23.4 (7.8–30.7)	0.514
OEA	65.5 (21.1–82.9)	49.5 (47.2–67.0)	48.7 (25.2–98.8)	47.7 (9.2–121.1)	0.674

*2-AG, 2-arachidonoylglycerol; AEA, N-arachidonoylethanolamine; PEA, N-palmitoylethanolamine; OEA, N-oleoylethanolamine*.

**Figure 2:**
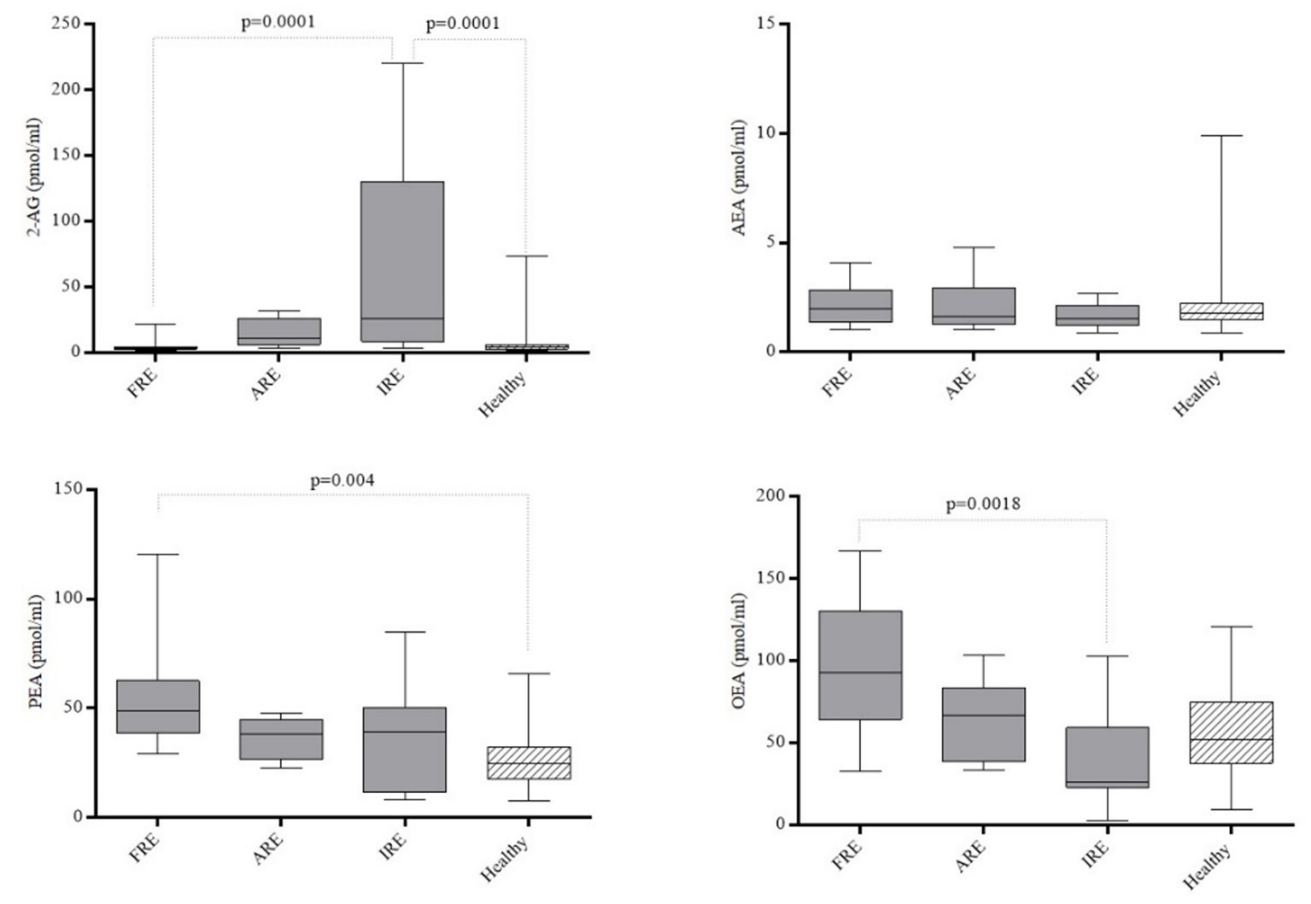
Plasmatic concentrations of 2-arachidonoylglycerol (2-AG), N-arachidonoylethanolamine (AEA), N-palmitoylethanolamine (PEA) and N-oleoylethanolamine (OEA) in healthy dogs and in dogs affected by Food-responsive Enteropathy (FRE, *n* = 10), Antibiotic-responsive Enteropathy (ARE, *n* = 9) and Immunosuppressive-responsive enteropathy (IRE, *n* = 14). Boxes show interquartile ranges with median values represented by the central lines, and minimum and maximum values represented by the endpoints of vertical lines.

Taking into account the different forms of CE (Figure 2), PEA levels were increased in the FRE group compared to healthy dogs (*p* = 0.04), while 2-AG was higher in IRE than in healthy dogs (*p* = 0.0001). Dogs affected by FRE also showed decreased 2-AG (*p* = 0.0001) and increased OEA levels (*p* = 0.0018) compared to IRE dogs. The plasma levels of the AEA and PEA in dogs with FRE, ARE or IRE did not show statistically significant differences.

**Figure 3:**
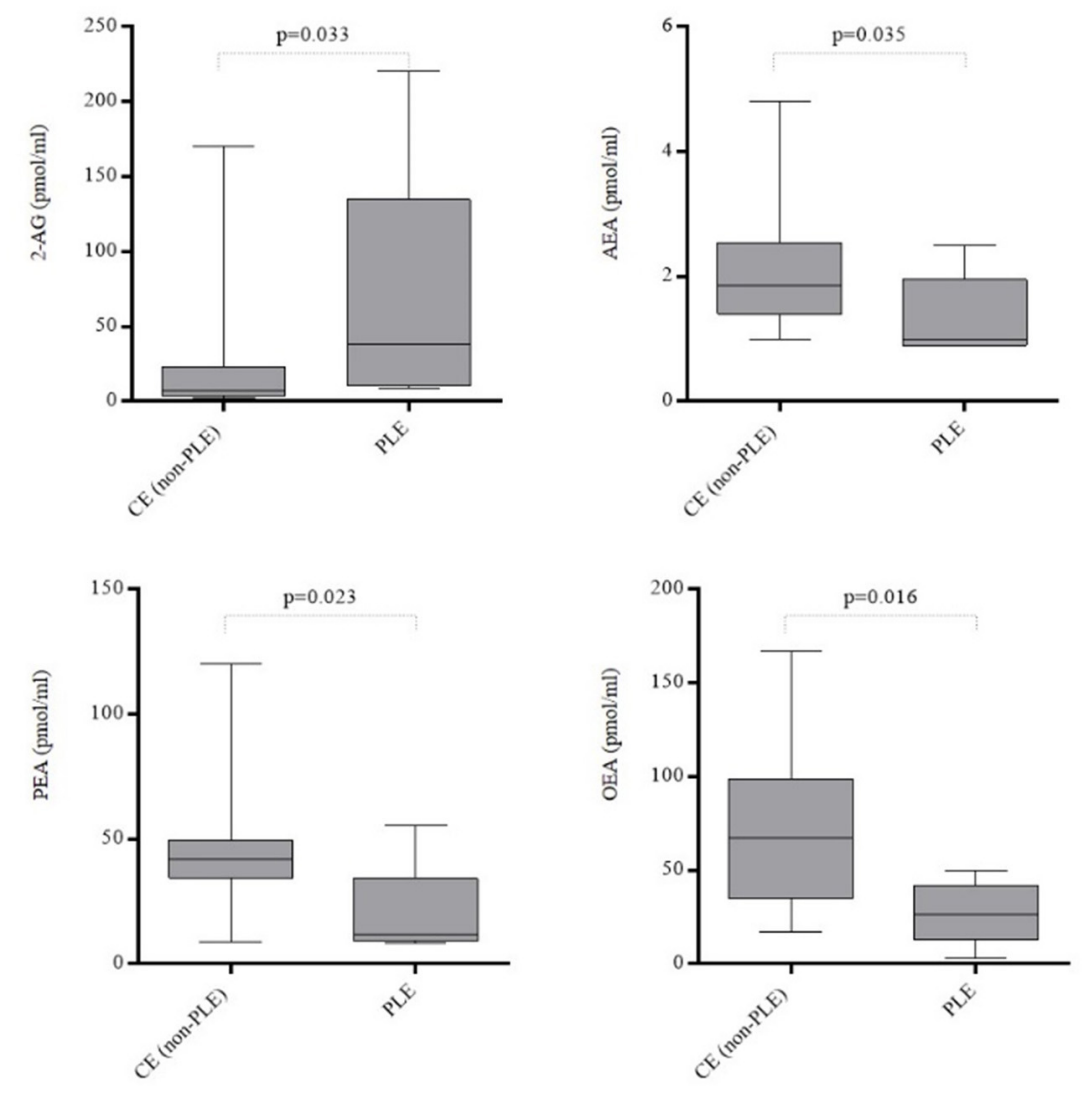
Plasmatic concentrations of 2-arachidonoylglycerol (2-AG), N-arachidonoylethanolamine (AEA), N-palmitoylethanolamine (PEA) and N-oleoylethanolamine (OEA) in dogs affected by chronic enteropathy with (*n* = 5) and without (*n* = 28) losing of proteins across the gut. CE, Chronic Enteropathy; PLE, Protein-Losing Enteropathy. Boxes show interquartile ranges with median values represented by the central lines, and minimum and maximum values represented by the endpoints of vertical lines.

Dogs with PLE showed increased levels of 2-AG (*p* = 0.033) and decreased AEA (*p* = 0.035), OEA (*p* = 0.016) and PEA (*p* = 0.023) when compared with those dogs affected by CE without losing of proteins (Figure 3). The plasma levels of the circulating plasma eCBs in the different CE subgroups were summarized in Table 6.

**Table 6:** Median values, with minimum and maximum in brackets, of plasma endocannabinoids (eCBs) in dogs with Food-responsive Enteropathy (FRE, *n* = 10), Antibiotic-responsive Enteropathy (ARE, *n* = 9), and Immunosuppressive-responsive enteropathy (IRE, *n* = 14).

**eCBs**	**FRE**	**ARE**	IRE^*^	***P*-value**	**CE (non-PLE)**	**CE (PLE)**	***P*-value**
2-AG	3.2 (1.9–22.2)	11.2 (3.3–31.5)	31.5 (3.9–220.6)	*<0.001*^*†*^	7.3 (1.9–169.9)	38.3 (9.0–222.6)	*0.033*
AEA	2.0 (1.0–4.1)	1.6 (1.0–4.8)	1.6 (0.9–2.7)	0.581	1.9 (0.9–4.8)	1.0 (0.9–2.5)	*0.035*
PEA	49.0 (29.3–120.3)	38.3 (22.5–47.6)	39.2 (8.0–85.1)	0.068	41.7 (8.8–120.3)	11.9 (8.0–55.4)	*0.016*
OEA	93.0 (32.8–167.0)	67.0 (33.8–103.6)	26.2 (2.8–102.8)	*<0.01*^*†*^	67.3 (17.2–167.0)	26.2 (2.8–49.8)	*0.023*

*Median values, with minimum and maximum in brackets, of plasma endocannabinoids (eCBs) in dogs with chronic enteropathy with (PLE, n = 5) and without Protein Losing Enteropathy (non-PLE, n = 28). CE, Chronic Enteropathy; 2-AG, 2-arachidonoylglycerol; AEA, N-arachidonoylethanolamine; PEA, palmitoylethanolamide; OEA, oleoylethanolamide*.

* *IRE group includes five PLE dogs*.

† *significant difference between IRE and FRE dogs. Italic denotes significance*.

Overall, in dogs affected by CE, including PLE dogs, regression analysis showed no correlation between plasma levels of eCBs and CIBDAI, CCECAI, CRP, folate, cobalamin or BCS.

**Figure 4:**
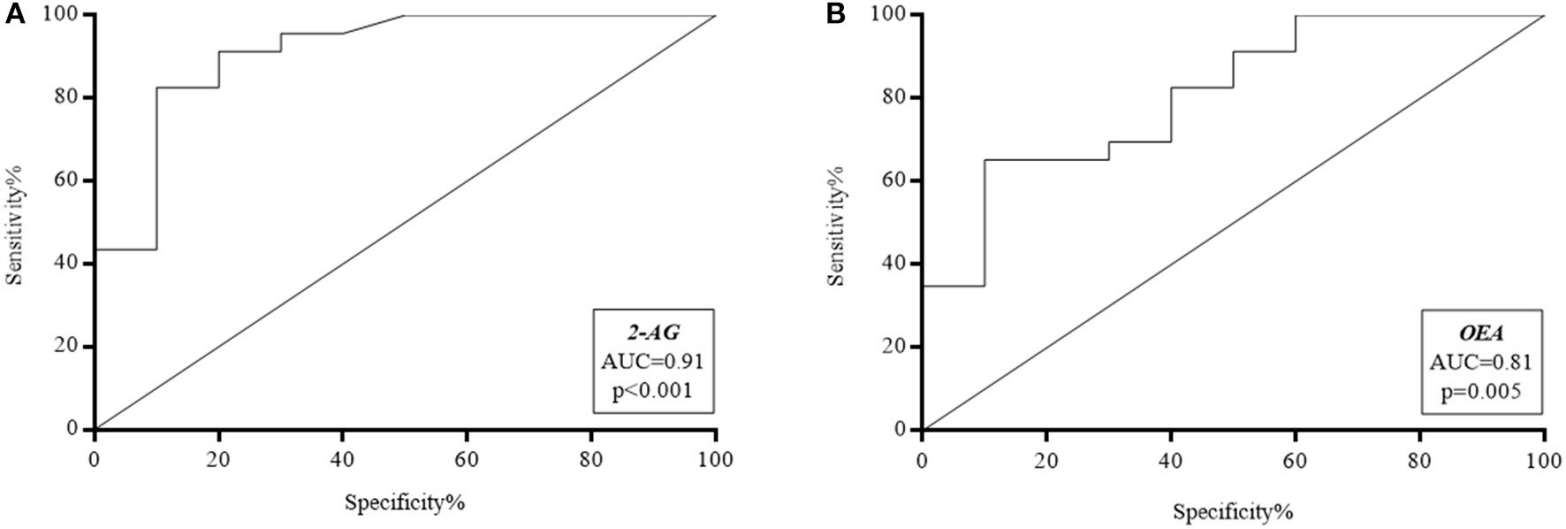
Receiver operating characteristic (ROC) curves of **(A)** 2-arachidonoylglycerol (2-AG) and **(B)** N-oleoylethanolamine (OEA). AUC, Area Under Curve.

In dogs with compatible clinical signs, the ability of eCBs to discriminate between FRE, ARE, IRE and PLE was interrogated through the area under the ROC curve (AUC) (Figure 4). An accuracy of 0.91 (95% confidence interval [CI], 0.79–1.03) was found for 2-AG, and of 0.81 (95% CI, 0.65–0.97) for OEA. In particular, values >3.2 pmol/ml for 2-AG showed 100% Se and 50% Sp in ruling out FRE, whereas a value >22.2 pmol/ml could exclude FRE with 100% Sp and 38.9% Se. The cut-off value of 6.5 pmol/ml showed 90.0% Sp and 82.6% Se, with a positive likelihood ratio (+LR) of 7.8. As for OEA, values <29.5 pmol/ml yielded the best Se (100%) with 34.8% Sp in excluding FRE, whereas values <108.9 pmol/ml showed the best Sp (100%) with 40% Se to rule out FRE rather than ARE or IRE. The cut-off value of 57.6 pmol/ml (Sp 90.0%; Se 65.2%) showed a +LR of 6.5. The diagnostic efficacy of the eCBs to detect/diagnose FRE rather than ARE or IRE is shown in Table 7.

**Table 7:** Diagnostic efficacy of 2-AG to diagnose ARE or IRE and diagnostic efficacy of OEA to diagnose FRE in dogs.

**2-AG pmol/ml**	**Sensitivity**	**95% CI**	**Specificity**	**95%CI**	**+LR**	**–LR**
≥1.9	100.00	81.5–100.0	0.00	0.0–30.8	1.00	
>3.1	100.00	81.5–100.0	50.00	18.7–81.3	2.00	0.00
>3.3	94.44	72.7–99.9	60.00	26.2–87.8	2.36	0.36
>3.8	94.44	72.7–99.9	70.00	34.8–93.3	3.15	0.18
>3.9	88.89	65.3–98.6	70.00	34.8–93.3	2.96	0.54
>4.2	88.89	65.3–98.6	80.00	44.4–97.5	4.44	0.49
>6.2	77.78	52.4–93.6	80.00	44.4–97.5	3.89	0.65
>6.4	77.78	54.4–93.6	90.00	55.5–99.7	7.78	0.51
>15.4	38.89	17.3–64.3	90.00	55.5–99.7	3.89	0.64
>22.2	38.89	17.3–64.3	100.00	69.2–100		0.56
>169.9	0.00	0.0–18.5	100.00	69.2–100		0.68
**OEA pmol/ml**	**Sensitivity**	**95% CI**	**Specificity**	**95%CI**	**+LR**	**–LR**
≥17.2	100.00	69.2–100.0	0.00	0.0–18.5	1.00	
>26.2	100.00	69.2–100.0	27.78	9.7–53.5	1.38	0.00
>32.8	90.00	55.5–99.7	27.78	9.7–53.5	1.25	0.36
>53.5	90.00	55.5–99.7	55.56	30.8–78.5	2.03	0.18
>65.3	70.00	34.8–93.3	55.56	30.8–78.5	1.57	0.54
>67	70.00	34.8–93.3	61.11	35.7–82.7	1.80	0.49
>67.5	60.00	26.2–87.8	61.11	35.7–82.7	1.54	0.65
>76.7	60.00	26.2–87.8	77.78	52.4–93.6	2.70	0.51
>84.4	50.00	18.7–81.3	77.78	52.4–93.6	2.25	0.64
>90.7	50.00	18.7–81.3	88.89	65.3–98.6	4.50	0.56
>101.6	40.00	12.2–73.8	88.89	65.3–98.6	3.60	0.68

*2-AG, 2-arachidonoylglycerol; OEA, N-oleoylethanolamine; CI, confidence interval; LR, likelihood ratio*.

## Discussion

The main goal of this investigation was to assess the potential of circulating eCBs as novel non-invasive biomarkers of canine CE, as well as their ability to discriminate even among different forms of the disease. Up to date, few studies investigated the alterations in plasma levels of eCBs in human chronic enteropathies.

In the present study, CE dogs showed increased plasma levels of 2-AG and PEA, compared to healthy dogs. Consistent with our results, in its investigation Fichna et al. (29) reported changes in plasma levels of 2-AG, PEA and OEA, but not of AEA, in patients with constipation-predominant and diarrhea-predominant Irritable Bowel Syndrome (IBS). On the other hand, recently, levels of AEA, PEA and OEA were found significantly increased in the plasma of patients with ulcerative colitis (UC) and Crohn's disease (CD) (30). Furthermore, plasma level of 2-AG was also increased in Inflammatory Bowel Disease (IBD) patients compared to controls (30). These results suggest that eCB signaling plays a key modulatory role in gastro-intestinal physiopathology, reflecting the pathological state of the inflamed intestine. It is possible to argue that chronic inflammation related to enteritis induces the release, from injured enterocytes and infiltrating leukocytes, primarily mast cells, together with eCBs (22, 29, 30), of several lipases, including soluble phospholipase A2, *N*-acyl phosphatidylethanolamine-specific phospholipase D and lysophospholipase D (31–33). Cell injury causes the increase in intracellular Ca^2+^ concentration, which stimulates *N*-acyltransferase producing *N*-acyl-phosphatidylethanolamines, the precursors of PEA and OEA. The generation of 2-AG may also be stimulated by Ca^2+−^dependent phospholipase C, the first enzyme in the 2-AG biosynthesizing pathway. Moreover, it is also possible that these enzymes, due to loss of integrity of the intestinal barrier, may enter the portal system and reach the blood, where they could catalyze the synthesis of 2-AG, PEA and OEA from membrane phospholipids of blood cells and endocytes (34, 35). Finally, activated macrophages, platelets and T and B lymphocytes release eCBs, providing an additional source of circulating eCBs (36–38). In addition, CB1 content have been implicated in dysbiosis-induced increases in intestinal permeability, inflammation, and modulation of the microbiota composition (39, 40).

To the best of our knowledge, this is the first study that interrogates the reference intervals of AEA, 2-AG, PEA and OEA in canine plasma in a cohort of owned healthy dogs, suggesting that gender or neutering status does not affect them. Of note, this finding is in partial agreement with the previously reported data in healthy humans, where no gender differences were found in AEA, PEA and OEA concentrations, while higher 2-AG levels were detected in males compared to females (27). In comparison between healthy dogs and dogs with CE, no statistical differences were observed regarding age and bodyweight, while male dogs were over-represented in the CE group compared to the control group. Despite no definite sex predisposition being reported in dogs with CE (41, 42), in a previous study more males than females were affected by CE (5).

The results of the present study confirmed that the clinical diagnosis of the different forms of CE is challenging and may present several pitfalls. For instance, FRE is usually considered more frequent in younger dogs, while IRE is often reported as an adult-onset disease (6). Incidentally, in the present study, the median age did not show significant differences among the dogs of the different CE groups. In the same way, the values of CCECAI and BCS did not seem able to distinguish different forms of CE, and their values significantly differ only in dogs with PLE. Also, in the present study, folate and cobalamin values did not significantly differ in dogs with FRE, ARE and IRE. Nevertheless, none of the dogs in the ARE group showed reduced cobalamin, as seen in previous literature on the low percentage of ARE dogs with such a pattern of folate/cobalamin (43). At histological examination the small intestine of all IRE dogs presented moderate to severe lymphoplasmacellular infiltrate. This finding is not surprising, with it being the most common change in CE dogs. Unfortunately, this parameter alone is unable to distinguish IRE from FRE or ARE (1, 3).

Given the above, it appears a relevant outcome of this study that, at the time of diagnosis, different forms of CE were associated with distinct profiles of eCBs in plasma. This supports the hypothesis that these circulating lipids hold promise for becoming candidate biomarkers for CEs, and possibly useful in predicting the most appropriate treatment. In particular, IRE dogs showed quite different eCB profiles when compared to animals that responded to the dietetic trial (i.e., FRE dogs), with increased 2-AG and reduced OEA levels in plasma. Other potential diagnostic biomarkers have been evaluated, however, only the serum CRP, the serum perinuclear anti-neutrophilic cytoplasmic antibodies (pANCA) and the fecal S100A12 showed similar ability in discriminate among the different form of CE with moderate to good sensitivity and specificity (7, 8). In particular, serum CRP was able to discriminate IRE or NRE from FRE or ARE with high sensitivity (72%) and specificity (100%) in one study (44). Whilst CRP is widely and routinely available, the high interindividual variability (45), together with the non-specific increases secondary to other inflammatory conditions (46), may affect its usefulness as CE biomarker, according to other studies (6, 47). Accordingly, CRP was herein found high in the sera of only four out of 17 dogs, regardless of the final diagnosis, without differences among the dogs affected by different forms of CE, including those dogs affected by PLE. In one study (48), the seropositivity for pANCA discriminated IRE/NRE from other causes of diarrhea, with a sensitivity of 51% and a specificity of 83%, while in other studies the pANCA was found higher in sera of dogs with FRE than in sera of those affected by IRE/NRE (Se 61–62%; Sp 77–100%) (49, 50). However, similar to serum CRP, seropositivity for pANCA can be also detected with other inflammatory conditions (51). In contrast to CRP and pANCA, the fecal S100A12 is a more specific marker of localized gastrointestinal inflammation (52), however, in a study, this protein showed a moderate accuracy in distinguish dogs with IRE/NRE from those with FRE or ARE, having a sensitivity of 64% and a specificity of 77% (26).

The determination of plasmatic 2-AG and OEA at the time of the first visit showed a good accuracy (AUC 0.91 and 0.81, respectively) in excluding FRE. In particular, cut-off values were identified (i.e., 2-AG levels >6.5 pmol/ml and OEA levels <57.6 pmol/ml) that may exclude FRE with a sensitivity of 90% and specificity of 82.6 and 62.5%, respectively, while 2-AG levels >22.2 pmol/ml and OEA levels <108.9 pmol/ml could exclude FRE with a sensitivity of 100%, in 38.9 and 40.0% of cases, respectively. These results, compared to the diagnostic performance of the above-mentioned biomarkers, suggest that circulating eCBs could have the potential to become a candidate biomarker in the diagnostic algorithm for canine CE.

On a practical point of view, these results gain further importance in the light of dilemmas recently roused in the scientific literature on use of antibiotics as diagnostic tool in canine CE. Indeed, the global concern for rising antibiotic resistance and the dysbiosis associated with indiscriminate use of antimicrobials suggest the necessity of avoiding empirical and injudicious use of these molecules in diarrhoeic dogs (44, 53, 54). In particular, despite circulating eCBs not seeming able to distinguish ARE from other CE forms, higher 2-AG and lower OEA may indicate a low likelihood for diagnosis of FRE. Recently, it was suggested to use antibiotics in chronic diarrhoeic dogs only at the end of the diagnostic protocol, once GI biopsies are performed, with evidence of infectious causes and in those cases showing signs of systemic inflammatory response syndrome (54). In the light of these recent advances and if one considers that, after the exclusion of extra-gastrointestinal and parasitic etiologies, the dietary trial is the first phase in the universally recognized step-up approach for diagnosis of canine CE, the assessment of circulating eCBs at the time of the first visit would be of further help in driving the most appropriate step in the approach to chronic GI signs in dogs.

Plasmatic eCBs did not seem to correlate with the severity of the disease (i.e., CIBDAI, CCECAI, CRP); yet, dogs showing protein loss across the gut (i.e., PLE), compared to those who did not, revealed a peculiar pattern of circulating eCBs, with an overall reduction of AEA, OEA and PEA in the face of increased 2-AG levels. It can be proposed that eCBs and their congeners play a role of controllers through a CB1-dependent mechanism (55) and *in vitro* studies showed that AEA might improve mucosal healing in patients with IBD (56), while a protective effect of PEA has been demonstrated in human biopsies from patients with active UC (57).

Moreover, different studies have shown that AEA and 2-AG increased *in vitro* intestinal epithelial permeability through CB1 (57–59), while it has been found that application of the eCB-like compounds PEA and OEA prevents the cytokine-induced increase in permeability and that knockdown of the same receptor had protective effects on epithelial permeability under cytokine-induced inflammation (15). Furthermore, in obese mice, antagonists of CB1 reduced plasmatic lipopolysaccharide levels in the bloodstream (39, 58, 60).

Since 2-AG is considered the major endogenous agonist of both CB1 and CB2 receptors that are involved in intestinal motility, secretion and inflammation and, even though the role of 2-AG in intestinal permeability remains controversial (59), the results of the present study appear of major interest. Indeed, PLE dogs usually have a more guarded prognosis compared to dogs with normal serum albumin concentration (5, 6) and further studies are warranted in order to interrogate the significance of systemic eCBs as prognostic marker of canine PLE and potential therapeutic target to manage this complex disease.

Specificity for the GI tract would be a desirable characteristic of an ideal biomarker for CE (7, 8) and non-specific increases in plasma levels of eCBs are detected in several disorders, especially those with a major inflammatory component, from acute pancreatitis (61) and atherosclerosis (62), to chronic hepatitis (63) and cirrhosis (64). This may potentially limit the clinical usefulness. Given this, eCBs levels should be tested at the beginning of the appropriate work-up for CE only after the exclusion of extra-gastrointestinal disorders.

A further potential limitation concerns the high overlap of circulating eCBs between diseased individuals from healthy controls and among the different forms of CE. It is unclear if these overlaps resulted from a biological variability or were secondary to a sub-clinical condition. To overcome this issue, a larger population should be investigated, with multiple specimens being collected over several days (7).

Besides, the long-term response is poor in ARE and IRE and the short duration of the follow-up could represent a limitation. Indeed, there are growing concerns that dogs classified as ARE and IRE when first diagnosed, will not respond longer term to the same degree (65).

In the light of this, and given the small number of CE dogs included, it appears clear that a larger study is mandatory to conclusively assess the impact of chronic enteropathy on circulating eCBs. However, in the present investigation clinically relevant differences in eCBs plasma concentrations were useful to support their potential as diagnostic biomarkers of distinct forms of canine CE. Additional longitudinal studies are also required to ascertain whether perturbations in eCBs levels occur over the course of the disease, and whether they are associated with particular presentations, rates of disease progression, response to therapy or prognosis. Finally, it would be important also to evaluate the state of expression and function of the different elements of the eCB system in the intestine (i.e., CB receptors, their ligands and eCB metabolic enzymes), in order to decipher the molecular and cellular mechanisms underlying the change of eCB tone (and signaling thereof) in CEs.

In conclusion, the present pilot study demonstrated that eCB signaling is altered in canine CEs, and that circulating levels of distinct eCBs may help to discriminate among the different forms of the disease in dogs. This also seems noteworthy from a practical point of view, because non-invasive and reliable biomarkers of CEs are currently unavailable. Indeed, diagnosis of canine CEs is usually complex and frustrating for both veterinary clinicians and owners. Therefore, evaluation of eCBs, as well as of their main congeners, may become a useful test in canine CE in addressing the diagnostic work-up, improving diagnostic performance and providing useful information regarding the potential target for treatment. It may also assist with reducing the time from referral to diagnosis and consequently reducing healthcare costs and psychological burden for the owners.

## Data Availability Statement

The raw data supporting the conclusions of this article will be made available by the authors, without undue reservation.

## Ethics Statement

The animal study was reviewed and approved by Committee on Animal Research and Ethics of the Universities of Chieti-Pescara, Teramo and Experimental Zooprophylactic Institute of AeM. Written informed consent was obtained from the owners for the participation of their animals in this study.

## Author Contributions

AB, AG, PEC, SO, TB, and MM: conceptualization. EF, GG, MP, FP, TB, GG, RDP, and PEC: investigation. SO and TB: methodology. SO, NB, PEC, and MDT: formal analysis. EF, PEC, SO, TB, MP, AG, AB, and MM: writing-original draft preparation and writing-review and editing. All authors have read and agreed to the published version of the manuscript.

## Conflict of Interest

The authors declare that the research was conducted in the absence of any commercial or financial relationships that could be construed as a potential conflict of interest.
