# Supplementary material for: Circulating Endocannabinoids as Diagnostic Markers of Canine Chronic Enteropathies: A Pilot Study
Source: Front Vet Sci. 2021 May 26;8:655311. doi: 10.3389/fvets.2021.655311 (PMC8187750; doi:10.3389/fvets.2021.655311)
Supplement: Supplementary file 1 [file Data_Sheet_1.pdf]

Supplementary Material

Circulating Endocannabinoids as Diagnostic Markers of Canine Chronic Enteropathies: a Pilot Study

Elettra Febo[1†], Paolo Emidio Crisi[1†], Sergio Oddi[1,2†], Marco Pietra[3], Giorgia Galiazzo[3], Fabiana Piscitelli[4], Alessandro Gramenzi[1], Roberta Di Prinzio[1], Morena Di Tommaso[1], Nicola Bernabò[5,6], Tiziana Bisogno[7*], Mauro Maccarrone[2,8*] and Andrea Boari[1*]

[1]Faculty of Veterinary Medicine, University of Teramo, 64100 Teramo, Italy

[2]European Center for Brain Research/Santa Lucia Foundation IRCCS, 00143 Rome, Italy

[3]Department of Veterinary Medical Sciences, *Alma Mater Studiorum*-University of Bologna, 40064 Ozzanodell'Emilia (BO), Italy

[4]Institute of Biomolecular Chemistry, National Research Council, 80078 Pozzuoli, Italy

[5]Faculty of Bioscience, and Technology for Food Agriculture and Environment, University of Teramo, 64100 Teramo, Italy

[6]Institute of Biochemistry and Cell Biology, National Research Council, 00133 Rome, Italy

[7]Institute of Translational Pharmacology, National Research Council, 00133 Rome, Italy

[8]Department of Biotechnological and Applied Clinical Sciences, University of L'Aquila, 67100 L'Aquila, Italy

[†]These authors have contributed equally to this work and share first authorship

*** Correspondence:**
Corresponding Authors
tiziana.bisogno@ift.cnr.it; mauro.maccarrone@univaq.it; aboari@unite.it

Supplementary Table 1. Breed, sex, age and bodyweight of the healthy dogs (n=30) and plasmatic concentrations of circulating endocannabinoids recorded for each healthy dog. M: male; F: female; Mn: neutered male; Fs: spayed female; 2-AG: 2-arachidonoylglycerol; AEA: N-arachidonoylethanolamine; PEA: N-palmitoylethanolamine; OEA: N-oleoylethanolamine.

Breed	Sex	Age (months)	Bodyweight (kg)	AEA (pmol/ml)	2-AG (pmol/ml)	PEA (pmol/ml)	OEA (pmol/ml)
English Bulldog	F	72	25	1.6	2.8	59.0	46.2
Jack Russell Terrier	Mn	78	7	2.4	2.2	65.9	47.2
Golden Retriever	M	6	23	1.7	1.6	34.2	52.4
American Pitbull Terrier	F	6	12	1.1	2.3	25.6	31.8
Golden Retriever	F	60	30	1.5	3.2	17.3	25.2
Mixed breed dog	Fs	66	27	2.1	6.1	19.2	51.9
English Bulldog	F	48	20	1.5	6.8	27.3	77.5
Mixed breed dog	Fs	96	18	1.6	4.4	23.4	76.5
Mixed breed dog	Fs	60	20	1.5	5.3	30.7	111.8
Dobermann Pinscher	F	22	35	0.9	4.5	22.8	39.5
Mixed breed dog	F	42	5	2.1	3.0	31.1	56.2
Jack Russell Terrier	F	54	6	1.5	3.6	19.2	70.2
Mixed breed dog	Mn	60	17	2.5	6.6	19.5	67.0
Jack Russell Terrier	Fs	48	5	2.0	4.0	12.0	31.9
Jack Russell Terrier	Mn	9	8	1.7	4.1	17.5	49.5
Golden Retriever	M	70	36	2.0	6.4	26.4	78.5
Beagle	M	72	16	1.3	4.3	17.5	64.1
Bernese Mountain Dog	Fs	24	35.5	2.1	4.6	23.4	46.6
Mixed breed dog	Fs	24	20	2.1	8.7	25.8	47.7
English Setter	Fs	102	21.7	2.4	2.5	30.5	121.1
Corso dog	M	60	59	2.6	2.7	22.6	54.0
Alaskan Malamute	M	24	24	1.8	3.0	31.6	66.9
Rottweiler	M	86	39.2	2.2	2.6	46.9	74.6
Siberian Husky	F	45	21	2.8	3.3	56.0	90.8
Siberian Husky	F	28	21.5	1.4	3.0	46.2	51.2
Mixed breed dog	M	12	5	2.8	2.4	57.1	82.9
English Setter	Fs	66	23	1.0	20.2	7.8	9.2
Jack Russell Terrier	F	24	5	1.3	24.8	11.4	26.7
Belgian Shepherd Dog	M	60	31	1.4	73.6	9.2	21.1
Border Collie	Fs	20	24	9.9	18.9	16.0	24.4

Supplementary Table 2. Comparison of sex, age and bodyweight between healthy dogs and dogs affected by chronic enteropathy (CE) (Mann-Whitney test or Fisher's exact test) and comparison of age and bodyweight among control group and dogs with Food-responsive Enteropathy (FRE), Antibiotic-

responsive Enteropathy (ARE) and Immunosuppressive-responsive enteropathy (IRE) (Kruskal–Wallis test and Dunn's multiple comparison post-hoc test). Italic denotes significance.

**Comparison**	**Age**		**Bodyweight**		**Sex**	
	Test	p value	Test	p value	Test	p value
Control *vs* CE	Mann-Whitney	0.14	Mann-Whitney	0.91	Fisher's exact test	*0.01*
Control *vs* FRE	Dunn's multiple comparison	0.08	Dunn's multiple comparison	0.22		
Control *vs* ARE		>0.99		> 0.99		
Control *vs* IRE		>0.99		0.96		
FRE *vs* ARE		0.08		0.17		
FRE *vs* IRE		>0.99		*0.02*		
ARE *vs* IRE		0.96		> 0.99		

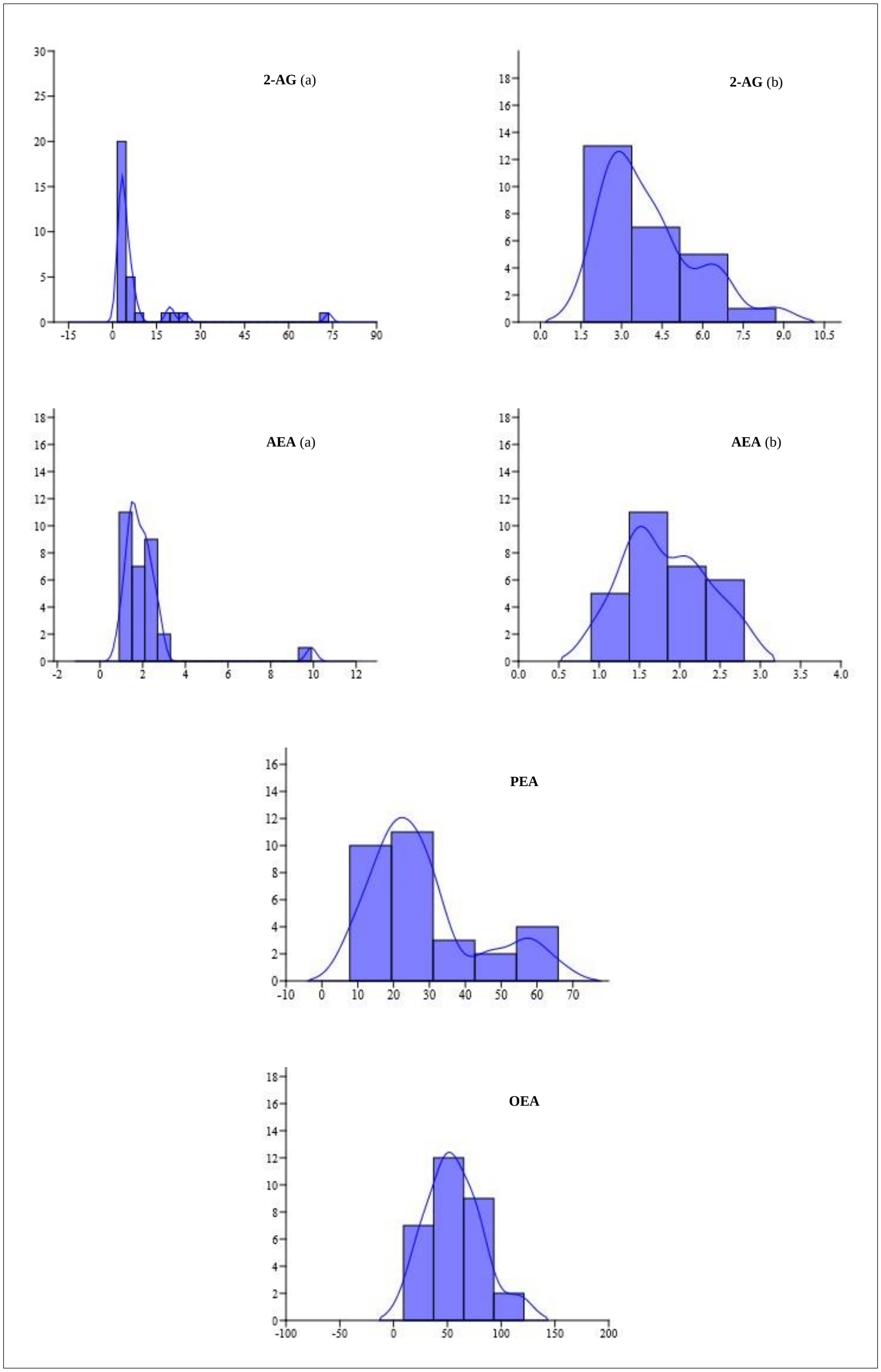

Supplementary Figure 1. Histograms of plasmatic concentrations of circulating 2-arachidonoylglycerol (2-AG) [including (a) or excluding (b) suspected outliers], N-

arachidonoylethanolamine (AEA) [including (a) or excluding (b) suspected outliers], N-palmitoylethanolamine (PEA) and N-oleoylethanolamine (OEA).
